# A framework for public involvement at the design stage of NHS health and social care research: time to develop ethically conscious standards

**DOI:** 10.1186/s40900-017-0058-y

**Published:** 2017-04-04

**Authors:** Raksha Pandya-Wood, Duncan S. Barron, Jim Elliott

**Affiliations:** 1 Faculty of Health and Life Sciences, De Montfort University, Hawthorn Building, The Gateway, Leicester, LE1 9BH UK; 2 Senior Research Fellow and Public Involvement Lead for the NIHR RDS South East, University of Brighton, Centre for Health Research, Falmer, Brighton, BN1 9PH UK; 3 Public Involvement Lead, Health Research Authority, Ground Floor, Skipton House, 80 London Road, London, SE1 6LH UK

**Keywords:** Ethics, Framework, Standards, Public involvement, Research design stage

## Abstract

**Plain English summary:**

Researchers who conduct studies in health and social care are encouraged to involve the public as early as possible in the process of designing their studies. Before their studies are allowed to start researchers must seek approval from a Research Ethics Committee, which will assess whether the study is going to be safe and ethical for patients or healthy volunteers to take part in. The process of ethical review does not consider how researchers work with patients and the public early on to design their studies. Furthermore, there is no requirement for researchers to seek ethical approval for public involvement. However, in our work advising researchers about public involvement we have found that the ways in which researchers involve the public in the design of their studies are sometimes unintentionally unethical, and this is the focus of our paper. We have observed ten areas where ethical issues may arise because of the actions researchers may or may not take and which might consequently have a negative impact. Therefore, we have used these observations to develop a “framework” to help researchers and the public work together at the early design stage in ways that are ethical. Our intention for the framework is to help researchers be mindful of these ten areas and how easily ethical issues can arise. The framework suggests some ways to overcome the potential issues in each of the ten areas. The ten areas are: 1) Allocating sufficient time for public involvement; 2) Avoiding tokenism; 3) Registering research design stage public involvement work with NHS Research & Development Trust Office at earliest opportunity; 4) Communicating clearly from the outset; 5) Entitling public contributors to stop their involvement for any unstated reasons; 6) Operating fairness of opportunity; 7) Differentiating qualitative research methods and public involvement activities; 8) Working sensitively; 9) Being conscious of confidentiality and 10) Valuing, acknowledging and rewarding public involvement.

We looked to see whether any other similar approaches to helping researchers address potential ethical issues when working with the public on designing studies have been published and to our knowledge none exist. Our framework is presented as a draft and believe that it would now benefit from input from researchers and the public to gauge how useful it is and whether there are any other possible situations that it might need to cover.

**Abstract:**

The current paper highlights real life examples of how ethical issues can arise during public involvement activities at the research design stage. We refer to “the research design stage” as the time between the generation of the research ideas and when formal permissions to start the work including ethical approval are granted. We argue that although most researchers work ethically at this early stage, some may still benefit from being informed about ethically conscious approaches to involving the public.

The paper highlights 10 ethical issues that we have observed with involving the public at the research design stage. We provide examples of these observed scenarios to illustrate the issues and make suggestions for how they can be avoided to help researchers become more ethically conscious when involving the public at the research design stage. Currently the draft framework comprises: 1) Allocating sufficient time for public involvement; 2) Avoiding tokenism; 3) Registering research design stage public involvement work with NHS Research & Development Trust Office at earliest opportunity; 4) Communicating clearly from the outset; 5) Entitling public contributors to stop their involvement for any unstated reasons; 6) Operating fairness of opportunity; 7) Differentiating qualitative research methods and public involvement activities; 8) Working sensitively; 9) Being conscious of confidentiality and 10) Valuing, acknowledging and rewarding public involvement.

The draft framework will help researchers to recognise the ethical issues when involving the public and is intended to be used voluntarily in a self-regulatory way. We believe that the draft framework requires further consultation and input from the wider research community and the public before endorsement by national UK bodies such as INVOLVE and the Health Research Authority (HRA).

## Background

### Public involvement work that has shaped the content of this paper

This paper is an opinion piece informed by the authors’ (RPW, DB) first-hand experiences of providing advice on involving the public as part of our National Institute for Health Research (NIHR) Research Design Service (RDS) roles. It also builds on consultations with approximately 20 lay people and 25 researchers during public involvement workshops held in the last 3 years in the East Midlands region. Prior to this, approximately 20 lay people and researchers gave input to early discussions on this topic at the 2010 INVOLVE national conference [1]. Finally, two lay people have read and commented on this paper prior to submission to the journal.

### Introduction

**Fig. 1:**
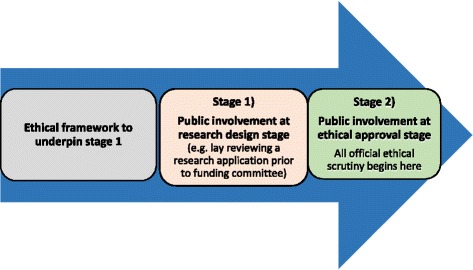
Promoting ethically conscious public involvement at the research design stage: the need for a framework. The above figure illustrates the stages of public involvement before a study is funded or approved to start. We believe an ethically conscious framework is required to help researchers understand the ethical issues that can arise prior to stage 1

Our experience as research advisers tells us that it takes researchers anything between six and twelve months or more to develop research applications. The “research design stage” we refer to throughout the paper concerns the time between the generation of research ideas right through to the point when formal ethical and other approval processes have begun (see Fig. 1 below) and includes involvement of the public from the earliest stage. The ‘Public involvement in research: values and principles framework’ [2] developed by INVOLVE [3], the National Institute for Health Research (NIHR) body advising on and promoting public involvement in England, states under their first principle ‘respect’ that: “Public members are involved in the ideas phase of the research, working with researchers and others to discuss priorities and research questions; public members help to make decisions about the research protocol and ethics application; public members review and provide feedback on grant applications” [2]. Therefore, the research design stage or “ideas phase” is a particularly valuable period for public involvement activities [4].

Such early public involvement activities have clearly reported impacts on, among other aspects, the research design and delivery [5] and add value to a study [6]. However, ethical issues can arise when involving the public at the research design stage and, in our experience, some researchers do not appear to be aware of them. We have used our experiences as research advisors to draft a framework to address these research design stage ethical issues.

Most of this paper has been shaped to address ethical issues concerning research applications being developed for national peer-reviewed funding competitions. We anticipate that the framework will be useful to any researcher.

We follow the definition of public involvement from INVOLVE: “doing research with or by people, not to, about or for them.” [7] What this means for health researchers is to meaningfully engage with people and to ask members of the public generally or those with lived experiences of a health condition and/or their carers for their input on research and then **acting** on that ‘lay’ feedback. When using the term ‘public’ we include patients, potential patients, carers and people who use health and social care services as well as people from organisations that represent people who use services [7]. By ‘ethics’ we mean “the rules of conduct and moral principles recognized in respect to a class of human actions or a group, culture, etc.” [8]. Similarly, ‘research ethics’ “refers to the moral principles guiding research from its inception through to completion and publication of results” [9], which can also be defined as the “fostering of research that protects the interests of the public” [10] and includes research participants and the researchers themselves.

We have drawn up a framework of 10 ethical considerations that can occur when researchers involve the public at the research design stage. These are intended to act as a checklist to help sensitise researchers and the public to ethically conscious working.

Our proposed framework has, after initial consultation, expanded from just three ethical issues in 2010 [1] to 10 issues in its current form, but we acknowledge it still may not be exhaustive. We are inviting input from the entire readership, particularly the public and researchers to help further develop and clarify the framework and related suggested approaches to addressing the issues.

## Main text

### Public involvement policy

The growth in public involvement across UK public services and health and social care research is linked to the increase in policy initiatives around citizenship, democracy and rights [11]. In the last decade, public involvement has become a key requisite in applied health research in the UK [12] and the commitment to this policy has been operationalised by the UK government in many ways. For example, the context for public involvement in the NIHR is shifting rapidly from health and social care reform to changes in the way research is conducted. There is also a growing awareness within the healthcare industry of the benefits that public involvement can bring to their research, resulting in increased involvement reported in applications for ethical review in recent years. However, the level is still very low compared to non-commercially funded research [13].

The Breaking the Boundaries strategic review of public involvement in the NIHR in 2014 resulted in the Going the Extra Mile (GEM) [14] report. This highlighted 11 recommendations for new ways of working in public involvement but none cover ethical conduct at the research design stage. At an operational level, our frontline experience of research design work suggests that ethical issues can and do arise at the research design stage. Sensitive and ethical working is required to ensure that research design stage activities are rooted in good practice and informed by first-hand experiences of the public who have been involved at this stage.

### Statutory policies relating to the ethical conduct of health and social care research

The Health Research Authority (HRA) and the Devolved Administrations for Wales, Scotland and Northern Ireland have consulted on and will shortly be issuing a new UK Policy Framework for Health and Social Care Research [15] which will replace the Research Governance Framework for Health and Social Care [12]. The new policy framework (shortly to be introduced) sets out the high-level principles of good practice in the management and conduct of health and social care research in the UK, as well as the responsibilities that underpin high-quality ethical research. Part of this is the requirement for health and social care researchers conducting most types of research in the NHS to submit their study for ethical review by an NHS Research Ethics Committee (REC) within the UK Health Departments’ Research Ethics Service. The Research Ethics Service (RES) is a core function of the HRA and is committed to enabling and supporting ethical research in the NHS. The RES is not responsible for university research ethics committees, although they work to similar standards and governance procedures, and they are not included in what we refer to as ethical or REC approval in this paper. The RES has two functions: 1) to protect the rights, safety, dignity and wellbeing of research participants and 2) to facilitate and promote ethical research that benefits participants, science and society [16]. The HRA clearly value public involvement stating that “involving patients and the public in health research will improve it by ensuring it is relevant to the needs of patients and more likely to have an impact on their health and wellbeing.” [17] (para B2; p.14).

The current Research Governance Framework and new policy frameworks are the only policies that cover what researchers do in designing new research studies (through the responsibilities of chief investigators, research teams, sponsors and employers). However, they do not contain anything specifically focused on how researchers work with the public to design and then conduct their studies.

RECs review applications for research and give an opinion about whether the proposed research is ethical and will protect the rights, safety, dignity and wellbeing of research participants. RECs also consider the safety and well-being of researchers and whether they have the relevant skills and experience to both conduct the research and to assess and deal with possible risks. Any ethical issues related to how researchers work with the public will be considered with respect to how the proposed research will be conducted rather than how the public were involved in designing it.

Ethical approval should in theory only be granted if everything that has led up to the application has been done in an ethically acceptable way. However, ethical review happens quite late in the research cycle, after funding has been approved, and the process focuses on what the research will do and not on how it was designed. We know through our own work that ethical, good practice and governance issues can, and do arise early on at the research design stage. REC approval is granted on the understanding that ethical principles are understood by researchers throughout the research cycle, but we are concerned that there is no existing specific policy or guidance relating to public involvement at the research design stage.

### Guidance relating to public involvement in research and research ethics committee review and potential areas of confusion

In May 2016, the HRA and INVOLVE issued an updated joint statement on public involvement in research and research ethics committee review highlighting that:

“You do not need to apply for ethical approval to involve the public in the planning or the design stage of research… even when those people are approached for this role via the NHS…” [18] (p.2).

There is an inherent risk that, when communicating that ethical approval for public involvement in the research design stage is not required, the message researchers may hear is that there are no ethical issues to be considered with respect to the public involvement at this stage. However, it is simply that public involvement activity in the research design stage does not fall within the statutory responsibilities of a REC, not that there are no ethical issues to consider. The point we make is that all formal good practice checks come only after funding is obtained rather than before and our concern is that sometimes ethical oversights might occur if the researcher is not mindful of the issues we highlight in the framework. Our observations from advising researchers at the research design stage suggest that ethical issues can occur in health and social care research much earlier than when a decision is made to fund the study.

There may also be researchers who are concerned that having close contact with people during the research design stage may by default require ethical approval since this level of contact with the public (who are also patients) may be no different to collecting research data. It is possible that some researchers are unaware of the HRA and INVOLVE joint statement. The update and clarification of the statement by the HRA and INVOLVE are, in part, welcome but wider communication may still be necessary, particularly, that ethical approval is not required at the research design stage.

Our proposed framework intends to clarify this grey area and could, once it has gained wider consultation and acceptance, be endorsed and signposted by the HRA and INVOLVE. If the framework is endorsed in this way and becomes widely and voluntarily used in a self-regulatory way, there is no need for a new formal process for reviewing public involvement in the research design stage.

The early involvement of the public at the research design stage is regarded by many as being valuable [5] and is clearly valued by INVOLVE and the HRA. For example, the briefing note that was issued to accompany the new HRA and INVOLVE joint statement on the ‘impact of public involvement on the ethical aspects of research’ [19] stresses that:

“Public involvement right at the beginning of a project helps researchers to identify new research topics and to modify their research questions…Involving the public early on…also helps researchers design and conduct their research in a way that potential participants consider to be ethically acceptable.” [19] (p.2).

However, while public involvement is recognised by INVOLVE and the HRA in helping to produce more “ethically acceptable” research there is no guidance pertaining to the ethical conduct of the research design stage concerning public involvement itself.

The new HRA and INVOLVE briefing note and joint statement highlights situations where the involvement of the public can pose ethical concerns. However, all the scenarios listed (e.g. helping to conduct interviews) relate to contact with participants or study data and would only happen once the study is funded and underway and not at the research design stage. Although researchers are required to detail in their ethical review application form how the public have contributed to the design of the study, the focus of ethical review is the future operation and conduct of the research and on “…how the public *will be* involved in…the research” [18] (p. 2, italics added), rather than how they *have been* involved at the research design stage.

The public involvement activities we focus on at the research design stage include the involvement of the public in the ‘design and development of research’, which is not covered by ethical review. This is partly because of the REC focus on how the research will be conducted and partly because there is rarely any detailed information included in an application that would give a REC cause for concern about the way that researchers have worked with the public. The HRA and INVOLVE joint statement says that “Involving the public in the design and development of research does not generally raise ethical concerns” [18]. However, our proposed framework currently highlights 10 potential areas related to the research design stage where we have observed ethical issues and which are related to the ways that researchers work with the public. There may well be others we have yet to be made aware of via further consultation.

### Differentiating between public involvement activities and qualitative research methods

In our experience, some researchers are unclear about the distinction between qualitative research methods and public involvement activities. It is possible that if researchers believe they are undertaking “interviews” when they are actually consulting the public at the research design stage then they may, erroneously, believe they require ethical approval. Defining and referring to information collected during public involvement consultations as “data” [20] is not very helpful and ethically is confusing. The use of language to refer to the research design stage is a point we address in the proposed framework and should help allay these concerns. Conscious attempts by researchers to deliberately combine qualitative research methods with public involvement early on [20] are, we believe, misguided. Any apparent “interface” between public involvement and qualitative research would be best acknowledged but kept separate. Engaging the public as research participants and treating their verbal contributions as data would always, in our minds, necessitate the gaining of ethical approval. This would, we believe, also apply if the information gained from consulting the public in the research design stage is presented as qualitative research data. The next iteration of the INVOLVE/HRA joint statement could be broadened to clarify these issues. We think this would be helpful.

In addition, the potential may be heightened for the public to be confused (or misled) at any time regarding their roles. Are the public always clear, for example when public involvement and qualitative research methods are being combined? More importantly are they clear when they are contributing through a public involvement role and when they are engaged as research participants? Researchers need to be clear about their purpose in consulting the public. If it is to acquire information to answer, in some way or other, their research question (s) then what they are doing is research and it needs ethical approval. However, if it is to help them design, conduct and or manage their study then that is involvement and does not need ethical approval.

### The literature supporting our case

We scoped the available literature for guidance explicitly addressing good practice (or what we refer to as ethically conscious) public involvement at the research design stage but we could not find anything. This adds support to our view that the issues we raise are being overlooked. However, we found four papers that discussed good practice concerning ethical issues related to public involvement in general.

Eight ethical considerations when involving the public in research were devised by Wright et al. [21] and accompanies their critical appraisal guidance as a standalone resource (p. 365). This resonates with good public involvement practice, but was designed for use by funders or journal reviewers (i.e. once the public involvement activities have already taken place). They were not intended, as far as we are aware, to specifically help guide the development of the research design stage as we are suggesting with our framework. However, we acknowledge useful elements contained in Wright et al’s [21] ‘ethical considerations’ but we feel the research design stage still requires its own ethical focus. Wright *et al*’*s* [21] guidance is being further developed to include assessments of funding proposals including ethical issues in research design [22].

Various public involvement toolkits exist providing advice on ways of involving the public in research (see Bagley et al., 2016 for an overview) [23]. For example, the Guidance for Reporting Involvement of Patients and Public (GRIPP) [24] checklist was developed to assist authors and reviewers in writing and assessing public involvement papers and reports (i.e. once the study is complete). It does not include an assessment of ethical issues, which may be addressed in its successor, GRIPP2 (yet to be published). The Public Involvement Impact Assessment Framework (PiiAF) [25] was designed to help researchers consider and work through a plan to capture any impact of public involvement. Researchers are guided to consider the values around public involvement in research at interpersonal, organisational and societal levels which are likely to impact on good practice at the research design stage (including issues of respect and trust). However, there is no direct reference to ethical considerations within PiiAF.

INVOLVE’s Values and Principles Framework [2] covers six areas for organisations, researchers and the public to consider for involvement at any stage of the research. While reference is made within the framework to the research design stage or “ideas phase” of research, it is meant for use across the life cycle of research. Brief mention is made of the need to involve the public from the outset (including being involved in the protocol development and ethics application) and to acknowledge their contribution. While the values and principles framework contains much of value only a few of the values resonate with the best practice we are proposing. Uniquely, our framework focuses solely on the research design stage, or “ideas phase”, which we believe requires quite separate and specific attention.

Also worthy of consideration here are two further papers. The first, the current consultation on ‘Standards for Citizen Science’ drawn up by a network of European universities [26], proposes that researchers ensure the rights and welfare of the public at any stage of their participation which can include: “…topic selection and development, research design, execution, dissemination of results and funding.” [25] (p.3). The second paper sets out ethical guidelines for patient groups contributing evidence to the development of health technology assessments [27]. These guidelines cover elements of our proposed framework but are not focused on the potential ethical issues that researchers might encounter when involving the public at the research design stage. Both papers appear relevant to our own framework and we intend to stay informed of their development.

The above literature helps to contextualise our proposed framework and highlights its unique position in the discussions regarding the research design stage. We know that there is a gap in dealing with ethical issues at this stage and our framework complements existing information. Our concern is that ethically conscious approaches to public involvement at the research design stage require clarity. There is little value in attempting to replicate other work that overlaps with our own. Our proposed framework could be readily incorporated into existing public involvement toolkits that aim to aid the planning, support and evaluation of public involvement and could be sign-posted as an additional resource within other documents or toolkits, (e.g. in the pilot clinical trials public involvement toolkit [23]). Website links to other useful resources within our proposed framework have been included (see Table 1). Feedback on additional useful resources that could be signposted within our framework would be welcomed.

**Table 1:** An ethically conscious framework for public involvement at the research design stage

Suggested Order	Areas of concern for Public Involvement at the Research Design Stage	Potential Ethical Issues for Involving the Public at the Research Design Stage	Suggested Solutions, including any related guidance that is available.
1).	Allocating sufficient time for public involvement	Inadequate allocation of time could result in public involvement not having the fullest impact upon the funding application and may contribute to stress and burden felt by those involved.	Building in realistic timelines, including at the research design stage: for example, offering the public involved at least two weeks to read and feedback on a funding application.
2).	Avoiding tokenism	When preparing a funding proposal public involvement activities at the research design stage are only listed in a ‘tick box’ (e.g. Stage 1 of the RfPB application process) without providing details anywhere else in the application.	Make any public contributions visible. Detail throughout the research application how the public have been involved in the research design stage and what difference it made. Who was involved? What did they do and contribute? How exactly did their input help shape the research proposal?
			Funding bodies to provide guidance on how and where research design stage public involvement activities could be “woven” throughout the application form? (e.g. how to supplement the public involvement tick box information for NIHR RfPB stage 1).
3).	Registering of research design stage public involvement work early with NHS Research and Development (R&D) Trust Office	Trust R&D offices not being informed of research design stage public involvement funding (e.g. RDS Public Involvement Fund) and that involvement of the public may sometimes occur on NHS premises (which may raise indemnity issues).	INVOLVE recommend that organisations and researchers include the governance of public involvement in their research accountability. Trust R&D offices can provide practical support around local governance arrangements.
4).	Communicating clearly from the outset	Not communicating clearly about public involvement roles and expectations at the research design stage can lead to disengaged and disenfranchised members of the public, unable to contribute to the study.	The public involved at the research design stage require the same information as the rest of the research team, but communicated in ways that they can access and understand.
			Use plain English to inform the public about the study and public involvement roles and responsibilities (verbally and/or print) to help people make informed decisions about becoming involved in the first instance (including research design stage). This is more likely to help them to be well informed and remain engaged.
		In consultative activities some group members can dominate if the consultation is not facilitated skilfully leading to some people not being able to communicate their ideas.	
			**See also**: Bagley et al.(2016) [23] Appendices 5&6: doi/10.1186/s40900-016-0029-8
			Wright et al. (2010) [21] Table 2. P.365: doi/10.1111/j.1369-7625.2010.00607.x :
			Consider group facilitation skills training to help manage dominant group members in order to capture the range of public involvement contributions.
			Some RDSs offer a consultation facilitation service in addition to public involvement funding.
5).	Entitling public contributors to stop their involvement for any unstated reason (s)	In order that the public who get involved do not become overwhelmed by what they are asked to do	Inform lay people early on that they can cease their involvement activities (including research design) at any time and without detailing their reasons for so doing
			**See also**: Wright et al. (2010) [21] Table 2. P.365: DOI: 10.1111/j.1369-7625.2010.00607.x
6).	Operating ‘fairness of opportunity’	Not taking issues around diversity and inclusion into account at the research design stage may result in disempowering, discriminatory research.	Respecting and valuing all forms of difference in individuals.
			If a research question is specific to a particular group or community, it is in the researcher’s interests to specifically engage and consult that community at the research design stage.
7).	Differentiating between public involvement activities and qualitative research methods	Reference to research design stage public involvement activities (e.g. consultations) using research terminology can confuse those who get involved about whether they are helping design the research or are participants in the research. It may also lead to conflicts of interest.	Avoid research terminology (e.g. ‘focus groups’) when referring to consultative public involvement activities.
			Do not combine qualitative research methods and public involvement activities; keep them separate. There is no reason why those who are involved in a study cannot also participate in some aspect of it but the difference needs to be made explicit. Researchers should also carefully consider whether this might present those involved with any potential conflicts of interest.
			Research Design Services (RDS) can help advise on the use of qualitative research methods and consistent practice and terminology in involving the public at the research design stage.
		Ambiguity around terminology may be linked to lack of clarity about exactly when ethical approval is required.	
8).	Working sensitively	Some of those involved at the research design stage may find the experience emotionally upsetting (particularly those with lived/carer experience of the condition under investigation). They may be reminded of negative health experiences or learn of risk factors or negative long term consequences of their condition for the first time. They may also become unwell making continued involvement difficult or impractical	Researchers need to avoid emotionally upsetting, sensitive situations and undertake public involvement sensitively at all times (including at the research design stage) especially with those with lived/carer experience of the condition under investigation and vulnerable people. Counselling or other support may be required. Additional time may be required if those involved are too unwell to perform their tasks.
			**See also**: Wright et al. (2010) [21] Table 2. P.365: DOI: 10.1111/j.1369-7625.2010.00607.x :
			Are service users well enough to participate? Will service users become distressed?
9).	Being conscious of confidentiality	Disclosure of personal, sensitive information during research design consultations (particularly when these take place in a group) can occur.	Ensure that a confidential environment is fostered at the beginning of every research design consultation. If the discussions are audio recorded, the reason for this and how the information will be used must be made clear. Permissions will need to be gained and confidentiality assured. See also area 7 above: if the information that was recorded is to be used to answer the research question in any way then this is research not involvement and ethical approval will be needed [18].
		Permissions to record the consultation may be overlooked.	
		Research ideas may be disclosed by public reviewers who have not been appropriately instructed about protocol confidentiality.	When the public are involved in reviewing protocols those doing so must adhere to a confidentiality agreement in the same way as any other reviewers.
			**See also**: Bagley et al.(2016) [23] Appendices 5&6: doi/10.1186/s40900-016-0029-8
			Wright et al. (2010) [21] Table 2. P.365: DOI: 10.1111/j.1369-7625.2010.00607.x
10).	Valuing, acknowledging and rewarding public involvement	Not valuing, acknowledging and rewarding the contributions of the public involved at the research design stage may lead to them feeling disempowered and marginalised.	Value and acknowledge public involvement at the research design stage by faithfully capturing contributions from the public in the research proposal and also via individual reward and recognition (i.e. payment; training opportunities)
			Out of pocket payments should be offered. Alert researchers to RDS Public Involvement Funding (available for research design consultations).
		There can be financial burdens for those involved at the research design stage, which if not addressed may deter people from getting involved.	
			**See also**: Bagley et al.(2016) [23] Appendices 5&6: doi/10.1186/s40900-016-0029-8
			Wright et al. (2010) [21] Table 2. P.365: DOI: 10.1111/j.1369-7625.2010.00607.x

Approaches to avoid ethical issues in involving the public in health and social care research prior to submitting for funding or receiving ethical approval (i.e. the research design stage)

### An ethically conscious framework for public involvement at the research design stage

The following 10 ethical issues relating to involving the public have been observed at the research design stage by the authors and confirmed in various public involvement discussions and workshops. We flag them as points of concern, and suggest that our accompanying framework could address each of them in turn. The issues we highlight are examples drawn from working with researchers or experienced by the public who have been involved at the design stage. The framework has been formulated to address potential ethical issues at the research design stage sequentially in the order that we think they are most likely to arise in practice. Each of the issues is illustrated with a scenario drawn from real situations we have observed. As indicated above, we looked for references to each issue grounded in established principles, frameworks or codes of conduct but were unable to find any that deal specifically with the relationship between professional researchers and public contributors. The sources we found are concerned with the relationship between either health and social care professionals and their patients or service users or between professional researchers and participants in their studies. However, we have cited principles we found that are transferrable and relevant to the relationship between professional researchers and public contributors. Feedback on the framework order and structure would also be welcome.

### Allocating sufficient time for public involvement

This idea of allocating sufficient time for public involvement in research ethics is not grounded in any previous ethical principles we have come across; however, we believe that this idea is embedded in the fundamental ethical principle of respect [2, 28]. The ethical issue we have observed: “*Can you ask two people in your group to read this by tomorrow. I am submitting the bid the next day*” (*Researcher*).

Allowing time for the recruitment of the public to take part in public involvement activities is crucial [5]. Time allocated for public involvement activities (e.g. when recruiting the public to take part in consultations and the time allocated for the length of those discussions) needs careful consideration. Allocating insufficient time for research design activities may suggest researchers are unlikely to incorporate meaningful input into their study and that they are not fully engaged or invested in public involvement. Inadequate allocation of time could result in public involvement not having the optimal impact on the proposal and may contribute to stress and burden felt by those involved [29]. For example, we feel that the public involved should be offered at least 2 weeks to review a proposal, so that they can fully think about their feedback. This resonates with INVOLVE’s support value highlighted in their Values and Principles Framework [2] and the need to build in realistic timelines to the research process, which we stress needs to commence at the research design stage.

### Avoiding tokenism

The issue of tokenism in research ethics concerning public involvement was raised in Ward et al., 2009 [30] as well as being embedded in the fundamental ethical principle of respect [28].

The ethical issue we have observed: “*The patients*’ *group didn*’*t really get what we are doing so I haven*’*t included their suggestions in the bid*.” (*Researcher*).

There are concerns that public involvement is frequently undertaken in a tokenistic manner [21]. Demonstrating involvement in funding applications is sometimes treated by researchers as unimportant and a ‘box ticking’ exercise [31, 32]. The new National Institute for Health Research (NIHR) Research for Patient Benefit (RfPB) 2 stage application process (which came in to effect in December 2015) in which public involvement is detailed at stage 1 in an involvement tick box is regarded by some as retrograde since no additional written evidence is required. In addition, researchers declaring that they have undertaken public involvement ‘consultations’ (i.e. one of the RfPB tick box options) may be treated by some as one of the ‘easiest’ of the three recognised ways (i.e. consultation, collaboration and user-led) in which people can be involved at the research design stage [33]. Choosing the ‘consultation’ tick box option should not be a convenient way of getting public involvement ‘brownie points’. One way of demonstrating good practice is by explaining in the research application how exactly research design public involvement has occurred and what difference it made to the proposal. Detailing who was involved and what they did along with exactly how the input was used, and how it shaped the research proposal should be woven throughout the relevant sections of a funding application form. Guidance from funders such as the NIHR on where and how to “weave” public involvement throughout the funding proposal to substantiate the tick box information would be useful.

### Registering of research design stage public involvement work early with NHS Research and Development (R&D) Trust Office

The registering of public involvement in R&D is a governance issue and we believe grounded in the fundamental ethical principles of safety [12, 15] and transparency [34, 35]. The ethical issue we have observed: “*I don*’*t need to inform my R&D office of my public involvement activity until the research gets funded*” (*Researcher*).

Early registering of research design stage public involvement work with NHS Trust Research and Development (R&D) offices should afford the researchers with practical and administrative support around local governance arrangements. R&D offices may be able to facilitate advice on NHS indemnity and whether specific statements apply to cover groups, such as non-employees or members of the public. However, ‘blanket cover’ should not be assumed (e.g. GPs are independent contractors and have their own indemnity providers). R&D offices are likely to be keen to be informed of any research design stage consultation funding awarded for public involvement activities (e.g. RDS Public Involvement Funds) since they will be responsible for the timely reimbursement of the public involved. INVOLVE recommend that organisations and researchers include the governance of public involvement in their research accountability policies (Value 6: Accountability) [2]. We believe these policies should be made explicit and include the research design stage. Early involvement and support from the Research and Governance infrastructure will also be valuable when planning public involvement as co-researchers throughout a study and the accompanying organisational and administrative issues this pose.

### Communicating clearly from the outset

The idea behind clear communication in research is grounded in the fundamental ethical principle of transparency [34], where research is open to public scrutiny through the publication of protocols and all results [35],. The ethical issue we have observed: “*I was pleased to be involved early on but I really did not understand what I was being asked to do. Not enough effort was made to explain things to me. I felt left out of the discussions*”. (*Public contributor*).

The public involved at the research design stage require the same information as the rest of the research team, but communicated in ways that they can access and understand. Potentially, not clarifying expectations early on can deter people from getting involved at the research design stage [36]. Details of the commitment required and potential burden of public involvement activities should also be communicated early on [5]. Providing adequate information about the study and about involvement roles and responsibilities verbally and/or in print is necessary and appropriate and helps people make informed decisions about becoming involved in the first instance and is likely to help them remain involved [5]. This may also include the necessity for clear communication early on about planned roles for the public as members of the research team (e.g. as co-researchers) in order to be clear about expectations later in the study and how people may require support to undertake these roles [37]. Linked to the issue of clear communication is a researcher’s need for facilitation skills (which differ to clinical interactions), to effectively capture all contributions when involving the public especially when some people may be more dominant than others [5].

### Entitling public contributors to stop their involvement for any unstated reasons

In research ethics, the idea of withdrawing from research is a well-grounded fundamental ethical principle for participants in studies and concerns respecting people’s wishes to stop doing something that they no longer wish to do [34, 38]. While the focus of that is on research participants the principle is equally relevant to public contributors or other members of a research team. The ethical issue we have observed: *A member of the public saying to a research colleague*, “*I have agreed to help design a study but I really feel overwhelmed by the amount of things they are asking me to do*, *I honestly haven*’*t got any more time and don*’*t want to continue but I feel like I have to help because I said I would* – *Initially they said it would be one meeting and so far I have had four two*-*hour long meetings with them*”. (*Public contributor*).

It is important that the public know that they can stop their involvement at any time and without detailing their reasons for so doing (in a similar way that applies to research participants). Following number 4 above, this may be a issue if roles and expectations have not been adequately communicated early on. It should be made explicit from the outset that the public can stop their involvement at any time.

### Operating ‘fairness of opportunity’

The value of fairness is enshrined in equalities legislation. Researching diverse groups and individuals falls within the fundamental ethical principle of respect [28], which includes equality of opportunity [35]. The ethical issue we have observed: “*My study is about young black and minority ethnic children but I can*’*t find any*.” (*Researcher*).

The backgrounds of who is involved at the research design stage are important to consider. If a research question is specific to a group or community, it is in the researcher’s interests to specifically seek to engage that community. Similarly, if the research question affects a broad group of people then the researcher needs to involve a broader group, considering inclusivity and diversity. INVOLVE define inclusivity and diversity as ‘respecting and valuing all forms of difference in individuals’ [39]. People differ in all sorts of ways which may not always be obvious or visible. These differences might include race and ethnicity, culture and belief, gender and sexuality, age and social status, ability and disability and use of health and social care services.’ [39] Awareness of issues concerning diversity and inclusivity demonstrates to people and the wider research team that the research being planned is to be conducted in an empowering and anti-discriminatory way. Diversity and inclusion are key challenges INVOLVE has highlighted in GEM that suggest that sometimes public involvement practice “…was perceived as being exclusive and not always fully meeting the requirements and goals of equality legislation.” [14] (p.39). Building inclusivity and diversity in to an ethically conscious approach to research design stage public involvement could help ensure that equality and anti-discriminatory practice is deeply rooted into the design of the research and therefore benefiting health for all. We have aligned this principle with GEM [14] replacing ‘diversity and inclusivity’ with ‘fairness of opportunity’ to better reflect these over-arching ideals. The issue of who best to include would need to also consider whether and when to include members of the public and/or those with lived experience of the condition the researcher is focusing on (e.g. when ensuring the relevance of the contents of a participant information sheet [40]). Consideration of matching the health experiences of the public involved with those of the target study participants therefore should be given, but in ways that do not disclose confidential or personal information in a stigmatising manner [40].

### Differentiating between public involvement and qualitative research methods

The blurring of boundaries between public involvement and qualitative research is documented as a methodological issue [41], but is not covered in any publications on ethics we are aware of. The ethical issue we have observed: “*Can I record and transcribe the data from my focus group with patients*” (*Researcher*) [*the researcher was referring to a public consultation meeting*].

Distinguishing between qualitative research methods and public involvement activities is often necessary at the research design stage. The use of terminology here is, we believe, important. Consultation or discussion groups are often referred to using qualitative research terms such as ‘focus groups’ and the information collected sometimes referred to as ‘data’. However, those involved at the research design stage are not research participants and so in our view it is not helpful to refer to these activities using such accepted research terminology. This ambiguity may be related to researchers being unclear whether REC approval is required to involve the public at the research design ethical stage (highlighted earlier). If data is collected and published this would necessitate REC approval since it is research.

Some researchers advocate the explicit and deliberate combining of public involvement activities with qualitative research methods [20]. As highlighted earlier, we feel that this is misguided and can lead to considerable confusion and so is not generally appropriate. Although there is no reason why those who are involved in a study cannot also participate in some aspect of it the difference between the roles and what is expected need to be made explicit. Researchers should also carefully consider whether this might present those involved with any potential conflicts of interest.

Participatory research is a valuable research method that starts with public involvement and ends with the public becoming co-researchers collecting, analysing and interpreting the data. However, there will, or at least should be, other public involvement throughout a participatory research project. There cannot be participatory research without involvement but it just adds to the widespread confusion to deliberately confuse or even combine involvement (which is a way of working between researcher and the public throughout a study from first concepts through to implementation of the findings) with a specific method for data collection and analysis.

The national network of RDSs can advise on the use of qualitative research methods and consistent and appropriate terminology and practice for involving the public in the research design stage.

### Working sensitively

The axiom ‘do no harm’ is one of the fundamental principles of ethics [28], and is also related to the notion of respect [34]. The ethical issue we have observed: “*I need to find two people who self*-*harm to sit on my public involvement group to help me plan the public involvement bits of my study*”. (*Researcher*).

Sometimes those involved at the research design stage may find the experience emotionally burdening if it reminds them of previously undisclosed, negative aspects of their (or someone they care for) health or experiences of care [42]. Researchers therefore need to involve the public sensitively especially with vulnerable people [43]. Care should also be taken by researchers not to disclose sensitive information (e.g. risk factors or negative long term progress or even death associated with a condition) which some people may not be aware of during early stage involvement. Sensitivity relating to a person’s own health and the impact it may have on their ability to be involved is also required [21].

### Being conscious of confidentiality

Research confidentiality and anonymity are well-established ethical principles [28, 34, 35] concerned with researchers needing to be mindful of an individual’s privacy. The ethical issue we have observed failed to take account of confidentiality: “*The study is on X*, [*removed the name of the sexually transmitted disease for confidentiality reasons*] *and Y* [*researcher uses a real patient*’*s name*] *has offered to be involved as the patient*” (*Researcher*).

Respecting the confidentiality of personal issues disclosed during research design stage consultation groups requires fostering a confidential environment. Permissions and confidentiality challenges may also need addressing if the public involvement consultations are audio recorded and stored. In addition, if the public are involved in undertaking protocol reviews prior to submission for funding this necessitates adherence to a confidentiality agreement in the same way as for any other reviewers.

### Valuing, acknowledging and rewarding public involvement

Researchers openly showing gratitude towards patients and the public in the research design process is not grounded in ethical principles but we believe forms part of the principles of respect [28, 34, 35] and integrity [34, 35] that will allow public contributors to contribute without undue personal burden, for example not being out of pocket because of their involvement. The ethical issue we have observed: “*It*’*s too expensive to involve people* [*at the research design stage*]. *I am not going to do it and anyway I don*’*t have the time*”. (*Researcher*).

Researchers should be encouraged to highlight how involvement at the research design stage has been incorporated into their study. Valuing people as individuals with expertise to contribute [5] will lead to meaningful involvement and reduce the likelihood of them feeling disempowered and marginalised [44]. There can be financial burdens for those involved at the research design stage [45] and so it is important to also acknowledge involvement through reward and recognition. Out of pocket expenses should always be offered. Funding for research design stage public involvement is offered by most RDSs [46]. Alerting researchers that such funds are available for public involvement would be beneficial. However, some people may not accept payment, preferring other rewards (e.g. training opportunities). This is similar to the respect value in the INVOLVE’s Values and Principles Framework [2], but again we recommend that it is stressed and applied at an earlier stage. Individual feedback to those who have been involved as well as communicating about the progress of the funding application are also important ways to value them [5].

## Conclusion

### Reflections on ethical research design stage public involvement practice

We are not proposing a new statutory approval process for public involvement at the research design stage, but envisage use of the proposed framework would be voluntary. Hence it will only be useful if there is broad input in to its refinement and wide awareness and adoption by the research community and the public. Discussions with peers and the public suggest that ethical sensitivity for public involvement at the research design stage is sometimes overlooked, indicating that this framework is very much necessary [1].

It should not be assumed that deliberately unethical practice in involving the public at the research design stage is common, but in our experience, it is sufficiently frequent to warrant us to propose the above framework to address poor practices. Some researchers may be unaware of the potential ethical challenges that can arise when involving the public at the research design stage. Alerting researchers and the public to ethically conscious approaches to public involvement at the research design stage would benefit their work and strengthen public involvement generally. If research design stage public involvement activities are conducted to consistent ethical standards by means of the proposed framework, then this would also enable the research community to better assess the impacts of public involvement on their research. This would enable the evidence base for the overall impact of public involvement at all stages of the research journey to be broadened and underpinned by an ethically strong foundation. In line with other recent calls, we would suggest that these assessments move away from quantitative measurement and consider context [4, 47].

The 10 ethical issues highlighted above form the basis of an ethically conscious framework for all researchers embarking on research design stage public involvement activities. These are summarised in Table 1.

### Moving forward

Our framework may not currently be exhaustive and so we would welcome further input from others, including the public, researchers, funders and policy groups. We recommend that a consultation or Delphi exercise (possibly utilising a Wiki governance type model to help facilitative broad consultation [48]) is conducted on the above 10 issues in our framework to explore whether there is consensus and whether there are any additional issues to consider. Input from funders and groups such as the HRA and INVOLVE would help with the framework’s dissemination and adoption. The public can often offer unique insights in to ethical issues arising in research [3, 20]. It is therefore crucial to gain further input from the public on the proposed framework so that it has wide relevance, application and adoption.

We believe that there is international transferability to this work; the concepts of research design ethics we raise, we hope are a universal part of any democratic country with research principles in health and social care. The next stage of this work will require input from international agencies across health, academia, industry and the general global public to help join forces to fully understand research design stage ethics involving the patients and the public. In connection to this, we understand limitations to our work, in that we are advisers/researchers based in the UK, however to offer a more rounded picture about the ethical issues we have raised, where possible we have consulted international literature to strengthen and support case.

The scope of this paper was based on experience of advising on UK peer-reviewed publicly funded competitions. However, we believe that the issues we raise are relevant to commercial funding too. The next phase of this work will include consultation with the healthcare industry.

In addition to the proposed Delphi consultation and to help shape the second stage of this work we invite responses to our paper via email to: hra.epird@nhs.net.

## Abbreviations

GEM: the National institute for health research’s going the extra mile report on public involvement in health and social care research
GRIPP: Guidance for reporting involvement of patients and public
HRA: the Health research authority
NIHR: the National institute for health research
PiiAF: the Public involvement impact assessment framework
R&D Office: NHS Trust research and development office
RDS: National institute for health research’s research design service
REC: NHS Research ethics committee
RES: UK Health departments’ research ethics service
RfPB: National institute for health research’s research for patient benefit programme
